# Structural Characterization of the Binding Interactions of Various Endogenous Estrogen Metabolites with Human Estrogen Receptor α and β Subtypes: A Molecular Modeling Study

**DOI:** 10.1371/journal.pone.0074615

**Published:** 2013-09-30

**Authors:** Pan Wang, Campbell McInnes, Bao Ting Zhu

**Affiliations:** 1 Institute of Zoology, Chinese Academy of Sciences, Beijing, P.R. China; 2 Department of Drug Discovery and Biomedical Sciences, South Carolina College of Pharmacy, University of South Carolina, Columbia, South Carolina, United States of America; 3 Department of Pharmacology, Toxicology and Therapeutics, School of Medicine, University of Kansas Medical Center, Kansas City, Kansas, United States of America; 4 Department of Biology, South University of Science and Technology of China, Shenzhen, China; Concordia University Wisconsin, United States of America

## Abstract

In the present study, we used the molecular docking approach to study the binding interactions of various derivatives of 17β-estradiol (E_2_) with human estrogen receptor (ER) α and β. First, we determined the suitability of the molecular docking method to correctly predict the binding modes and interactions of two representative agonists (E_2_ and diethylstilbesterol) in the ligand binding domain (LBD) of human ERα. We showed that the docked structures of E_2_ and diethylstilbesterol in the ERα LBD were almost exactly the same as the known crystal structures of ERα in complex with these two estrogens. Using the same docking approach, we then characterized the binding interactions of 27 structurally similar E_2_ derivatives with the LBDs of human ERα and ERβ. While the binding modes of these E_2_ derivatives are very similar to that of E_2_, there are distinct subtle differences, and these small differences contribute importantly to their differential binding affinities for ERs. In the case of *A*-ring estrogen derivatives, there is a strong inverse relationship between the length of the hydrogen bonds formed with ERs and their binding affinity. We found that a better correlation between the computed binding energy values and the experimentally determined log*RBA* values could be achieved for various *A*-ring derivatives by re-adjusting the relative weights of the van der Waals interaction energy and the Coulomb interaction energy in computing the overall binding energy values.

## Introduction

The endogenous estrogens are a group of vitally important female sex hormones with diverse biological functions. Disruption of their actions contributes to the pathogenesis of a number of disease states in humans, including disruption of reproductive functions [1]–[3], development of breast cancer [4]–[8], and many other abnormal conditions [9]–[10]. Undoubtedly, it is of considerable scientific interest to develop the ability to predict the estrogenic potency and efficacy of a given chemical. Compared to the widely used methods such as receptor binding assay, receptor activity assay and crystallography, computational molecular modeling methods have the potential advantages of low cost, high speed, and high throughput. Progress in this area of research will help develop the ability to predict and/or identify new environmental estrogens among millions of natural and synthetic compounds that humans are potentially exposed to so that the general public can be informed and thus avoid unwitting exposure to these chemicals.

There are mainly two categories of computational methods commonly used in studying the ligand-receptor interactions. One is the more traditional QSAR (Quantitative Structure-Activity Relationship) approach, and the other is the molecular docking approach. The QSAR approach relies solely on the structures of ligands to predict the three-dimensional properties of the receptor binding site as well as the potential binding affinity of a given unknown ligand. In comparison, molecular docking analysis is based on the three-dimensional structures of both the receptor binding pocket and the ligand to predict its preferred binding conformations in the receptor’s binding site. The relative binding affinity of a ligand can then be estimated by computing the binding energy values (Δ*G* or Δ*E*). These two approaches have their own advantages and disadvantages. Whereas the QSAR model usually can achieve a good prediction when the unknown test compounds are structurally very similar to the training set of compounds used, the molecular docking analysis theoretically is more versatile in predicting the binding conformations of structurally-diverse compounds.

**Figure 1:**
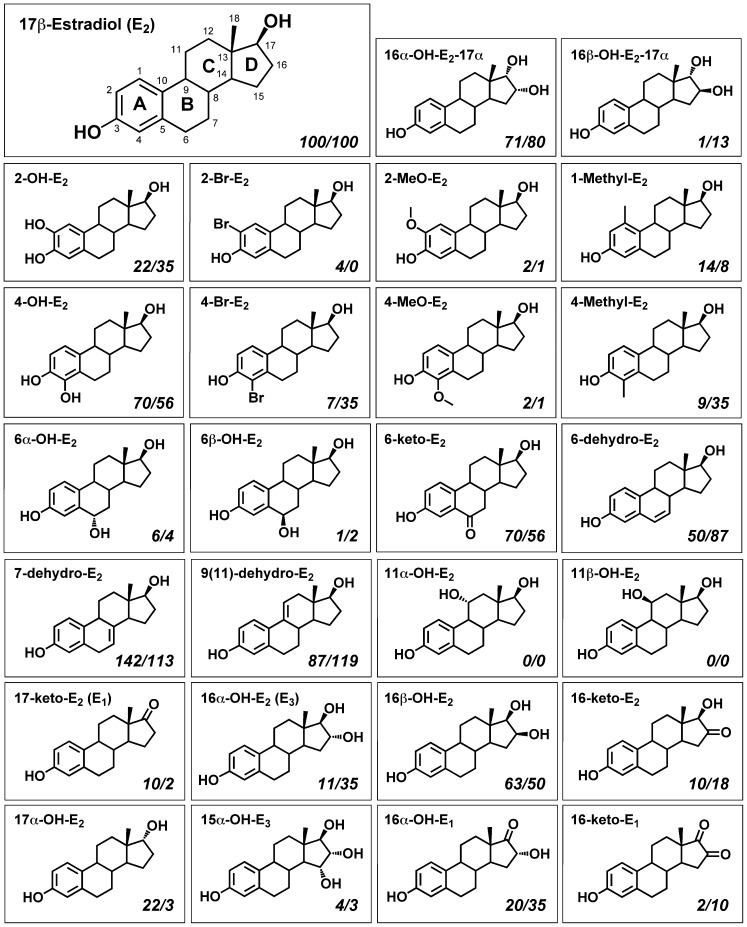
The chemical structures of 17β-estradiol (E_2_) and the 27 E_2_ derivatives. The number of each carbon is labeled next to the atom in the E_2_ structure. The names of the E_2_ derivatives were shown in the upper left corner of each frame. The *RBA* values of the E_2_ derivatives for ERα and ERβ (data from **Table 1** as % of *RBA* of E_2_) were shown in the lower right corner of each frame. The numbers were rounded to the nearest integer due to space constraint.

In an earlier study [11], we have compared the relative binding affinity (*RBA*) of some fifty estrogen derivatives (mostly the endogenous metabolites of 17β-estradiol, E_2_) for human ERα and ERβ. These estrogen derivatives, while sharing the same core structure as E_2_ with only one or two small functional group(s) added (their structures shown in **Figure 1**), have vastly different *RBAs* for human ERα and ERβ, ranging from having a similar binding affinity as E_2_ to having little or no binding affinity at all (see **Table 1**). In the present study, these E_2_ derivatives were used as model compounds to study their binding interactions with human ERα and ERβ using the molecular docking approach.

**Table 1:** Hydrogen bond lengths (Å) and calculated van der Waals interaction energy (Δ*E*_VDW_, kcal/mol) and Coulomb interaction energy (Δ*E*_Coulomb_, kcal/mol) between estrogen derivatives and the ERα and ERβ LBDs.

Estrogenderivatives names		ERα						ERβ					
		*RBA*	E353	R394	H524	Δ*E*_VDW_	Δ*E*_Coulomb_	*RBA*	E305	R346	H475	Δ*E*_VDW_	Δ*E*_Coulomb_
*A*-ring	E_2_	100	1.42	2.02	1.96	−53.37	−23.58	100	1.62	3.14	1.99	−52.06	−19.34
	2-OH-E_2_	22	1.46	2.06	1.94	−51.40	−35.32	35	1.64	3.14	2.04	−52.86	−25
	2-Br-E_2_	4	1.69	2.34	1.77	−39.88	−10.09	0.35	2.00	3.43	1.91	−40.03	−12.82
	2-MeO-E_2_	2.2	2.59	2.52	1.83	−37.31	−12.57	1.4	2.26	3.54	1.87	−36.46	−14.6
	1-Methyl-E_2_	14	1.60	2.03	2.0	−52.83	−16.04	8	2.05	3.30	2.05	−40.57	−14.05
	4-OH-E_2_	70.4	1.44	2.14	1.94	−51.32	−25.73	56	1.62	3.19	1.97	−52.86	−22
	4-Methyl-E_2_	8.9	1.42	2.24	1.87	−39.32	−22.95	35	1.79	3.42	1.83	−43.43	−18
	4-Br-E_2_	7.1	1.45	2.35	1.87	−38.74	−22.56	35	1.71	3.40	1.82	−38	−18.5
	4-MeO-E_2_	1.6	1.37	3.03	1.74	−24.34	−26.01	1	1.74	3.30	1.78	−30.57	−18.71
*B*/*C*-ring	6α-OH-E_2_	5.6	1.44	2.0	2.02	−50.8	−27.88	3.5	1.65	3.2	1.93	−50.55	−19.3
	6β-OH-E_2_	1.4	1.4	1.98	2.01	−47.37	−30.77	1.6	1.56	3.1	1.99	−50.96	−20.76
	6-Keto-E_2_	70.4	1.45	2.06	2.0	−52.26	−31.27	56	1.61	3.2	1.95	−50.88	−20.32
	6-Dehydro-E_2_	50	1.46	2.03	2.0	−51.17	−28.45	87	1.59	3.1	2.02	−52.8	−19.74
	7-Dehydro-E_2_	141.8	1.44	2.02	1.93	−51.08	−28.92	113	1.58	3.12	2.12	−54.79	−19.11
	9(11)-Dehydro-E_2_	87	1.44	1.98	2.03	−49.36	−27.85	119	1.61	3.09	1.98	−52.85	−19.82
	11α-OH-E_2_	0.01	1.44	2.01	1.87	−49.83	−29.41	0.01	1.63	3.14	1.94	−51.13	−22.09
	11β-OH-E_2_	0.01	1.44	2.01	1.97	−51.02	−27.44	0.01	1.62	3.14	1.98	−52.48	−20.26
*D*-ring	17-Keto-E_2_ (E_1_)	10	1.43	2.02	2.09	−50.48	−26.79	2	1.59	2.59	−	−51.17	−13.77
	16α-OH-E_2_ (E_3_)	11.2	1.43	1.99	2.01/2.0	−50.68	−32.45	35.4	1.59	3.11	2.05/3.81	−51.06	−16.69
	16β-OH-E_2_	63	1.43	1.98	2.01/2.0	−50.49	−30.35	50	1.56	3.05	3.09/1.98	−51.37	−21.67
	16-Keto-E_2_	10	1.41	1.94	2.09	−49.51	−28.06	17.8	1.70	2.6	2.01	−54.55	−14.89
	17α-OH-E_2_	22.4	1.44	2.02	2.86	−50.45	−25.21	3.2	1.58	3.07	3.43	−51.81	−16.36
	15α-OH-E_3_	4	1.43	1.98	1.98	−50.92	−34.71	2.5	1.58	3.11	2.05	−52.64	−18.09
	16α-OH-E_1_	20	1.43	2.01	2.08	−51.56	−31.27	35.4	1.6	3.07	–	−50.71	−18.34
	16-Keto-E_1_	1.8	1.41	1.95	2.11	−50.38	−27.18	10	1.54	2.52	–	−52.02	−11.63
	16α-OH-E_2_-17α	70.9	1.43	2.01	3.02/1.99	−51.44	−29.66	79.5	1.58	2.57	3.87/2.0	−51.67	−20.89
	16β-OH-E_2_-17α	0.9	1.43	2.01	3.45/1.96	−50.69	−28.67	12.6	1.55	3.01	3.74/2.02	−51.74	−21.52

Hydrogen bond lengths were quantified by measuring distances between the hydrogen atoms of the 3-hydroxyl group of estrogen derivatives and the O_ε_ of ERα-E353 or ERβ-E305, between the oxygen atoms of the 3-hydroxyl group of estrogen derivatives and the H_η_ of ERα-R394 or ERβ-R346 and between the hydrogen atoms of 17-hydroxyl group of estrogen derivatives and N_δ_ of ERα-H524 or ERβ-H475. For the *D*-ring derivatives, two hydrogen bond lengths were listed. The first is formed by hydrogen atoms of 17-hydroxyl groups and the second is formed by hydrogen atoms of 16-hydroxyl groups. Relative binding affinity (*RBA*) was also listed for comparison.

There are two main reasons that we chose to use these structurally similar estrogen derivatives as model compounds. First, although the crystallographic structure of human ERα in complex with E_2_ is known, the interactions of the receptor with various E_2_ metabolites are actually not known. Studying the interactions of these compounds with human ERs may shed light on how small modification in the E_2_ structure can drastically disrupt its binding interactions with the receptors. These results, in turn, may help us better understand the detailed structural characteristics of the binding interaction of an estrogen with various amino acid residues in the LBDs of human ERs. Second, we have successfully used the QSAR approach in an earlier study [11] to accurately predict the binding affinity of these structurally similar estrogen derivatives for human ERs. In the present study, therefore, we sought to use a molecular docking approach to predict the binding affinities of these estrogen derivatives for human ERα and ERβ, so that the predicted abilities of these two modeling approaches could be compared.

The research strategies used in this study are as follows: We first evaluated whether the molecular docking method can be used to reliably and correctly predict the binding interaction of a ligand with the ligand binding domain (LBD) of human ERα. Next, by using the validated molecular docking method, we then set to determine the binding interactions of a total of 27 representative E_2_ metabolites/derivatives (structures shown in **Figure 1**) with the LBDs of both human ERα and ERβ. Lastly, we also explored ways to achieve a better prediction of the binding affinity by modifying the calculation method for the binding energy (Δ*E*) values.

## Methods

### Building of the 3-D Structures of E_2_ Derivatives

For the ligands used in this study, their chemical structures and *RBA* to ERs are shown in **Figure 1**. As mentioned above, these estrogens share the same core steroid structure as E_2_; in fact, most of them are naturally occurring metabolites of E_2_ formed in humans [12].

Building of the ligand structures as well as energy minimization were performed using the *InsightII* modeling program (Version 2005, Accelrys Inc. San Diego, CA) installed in the Red Hat Enterprise Linux WS4.0 (Red Hat Inc. Raleigh, NC) operating system on a Dell Precision 690 workstation. The 3-D structures of these E_2_ derivatives were built with the *Builder* module in *InsightII* and minimized with the *Discover* module. Energy minimization was carried out with the Polak and Ribiere conjuate gradients method in the *Discover* module in *InsightII* until the final convergence criterion reached the 0.001 kcal/molÅ. Consistent valence force field (CVFF) was used for energy minimization.

### Selection of the Human ERα and ERβ 3-D Structures as Templates

The use of molecular docking approach to study the binding interaction of a ligand with its receptor protein is usually based on the known 3-D structure of the receptor protein. As listed in **Table 2**, presently about a dozen crystal structures of the ligand binding domains (LBDs) of the human ERα and ERβ are available in the PDB database [13]–[19], [22]. While some of the receptor proteins are in complex with agonists (such as E_2_ and DES), others are in complex with antagonists (such as raloxifene and tamoxifen). We compared the ERα structures in complex with three ERα agonists, namely, E_2_ (PDB codes 1ERE and 1A52), DES (PDB code 3ERD), and genistein (GEN) (PDB code 1X7R) by superimposing these structures on each other. The Root Mean Square Distance (RMSD), a parameter that is commonly used to reflect the average distance between the same atoms in different structures, was found to be only approximately 0.5 Å for these ERα structures when they were complexed with different agonists. By superimposing the ERβ structures in complex with two different ERβ agonists, E_2_ (PDB codes 3OLS) and ERB041 (PDB code 1X7B), their RMSD was found to be 0.6 Å. This indicates that the receptor structures resolved by different research groups under rather different experimental conditions have very similar overall structures. Therefore, in this study, the *x*-ray crystal structure of human ERα in complex with E_2_ (PDB code: 1ERE) [14] and the *x*-ray crystal structure of human ERβ in complex with a synthetic ERβ agonist ERB-041 (PDB code: 1X7B) [18] were used as templates for docking various estrogen derivatives. Hydrogen atoms were added to the proteins, and the structures were minimized to relax the strains imposed by the addition.

**Table 2:** Summary of current 3D structures of ERs in complex with various ligands (listed according to the chronological order of the publications).

ER isoform	Ligands	PDB code	References	Comments
ERα-LBD	E_2_	1ERE	[14]	The first steroid receptor structure with an agonist bound inside the LBD.
ERα-LBD	Raloxifene (RAL)	1ERR	[14]	The first ER structure with a SERM bound inside the LBD.
ERα-LBD	E_2_	1A52	[15]	A similar structure as 1ERE.
ERα-LBD	DES/peptide	3ERD	[16]	The first ER structure with an agonist and a co-activator peptide bound.
ERα-LBD	4-hydroxyl tamoxifene (OHT)	3ERT	[16]	In this structure the ligand in the LBD induces a similar orientation of H12 as seen in 1ERR.
ERβ-LBD	RAL	1QKN	[17]	The first ERβ structure with an antagonist bound inside the LBD.
ERβ-LBD	ERB041	1X7B	[18]	An ERβ structure with an agonist bound inside the LBD.
ERα-LBD	Genistein (GEN)	1X7R	[19]	An ERα structure with an agonist bound inside the LBD.
ERβ-LBD	GEN	1X7J	[19]	The ERβ structure with a partial agonist bound inside the LBD. GEN has 40-fold higher binding affinity for ERβ than ERα.
ERβ-LBD	E_2_	3OLS	[22]	An ERβ structure with an agonist bound inside the LBD.

### Molecular Docking and Simulation Studies

Molecular docking were performed using the *InsightII* modeling program (Version 2005, Accelrys Inc. San Diego, CA) installed in the Red Hat Enterprise Linux WS4.0 (Red Hat Inc. Raleigh, NC) operating system on a Dell Precision 690 workstation. The flexible docking procedures were carried out using the *Simulated Annealing Docking* method in the *Affinity* module of *InsightII*. The binding pocket in ERα was defined to include all residues within the 7-Å reach of the original ligand E_2_, which include M343, L346, A350, E353, L387, L391, R394, L402, F404, I424, H524, and L525.

A combination of *Monte Carlo* and *Simulated Annealing* methods was used to explore all possible conformations of a ligand molecule in the binding pocket. One hundred conformations were obtained and the one with the lowest potential energy was chosen for further minimization. The backbone of the protein was fixed during the docking procedures. Using similar protocols as described above for ERα, flexible docking of various ligands into the LBD of human ERβ was also carried out.

Detailed docking parameters are as follows. When we set up the docking run, the ligand binding site was defined, and the “solvation_grid” was set to “on”. Alpha carbons of the receptor were held fixed during the docking run. The *Affinity* docking method uses a combination of Monte Carlo (MC) and Simulated Annealing (SA) approaches. In the initial phase, an MC search generated 100 unique ligand binding orientations. The “quartic_vdw_no_coul” was selected as the “nonbond_method” because randomly placing the ligand in the binding pocket could potentially lead to very severe divergences in the Coulombic and VDW energies. The “vdw cutoff” was set to 5, the “scale_vdw” was set to 0.1, the “scale_HB” was set to 1, and the “scale_tether” was set to 1. In addition, the “TempOrERange” was set to 200, the “MxrChange” was set to 180, the “EnerToler” was set to 1e+6, the “RMS_Toler” was set to 1. Minimization step was set to 10,000.

After the initial structures had been generated by the initial MC phase, these structures were then read in as an “.arc file” for further refinement by SA. The SA phase consisted of 50 cycles of 100 fs molecular dynamics (MD) simulations. The temperature is re-scaled at each cycle, with the first cycle running at 500 K and the final cycle at 300 K. Following the SA phase, the structure is minimized by 10000 steps of conjugate gradients. The “Cell_multipole” was selected as the “Nonbond_method”. The “diel_const” was set to 1, the “scale_vdw” was set to 1, the “scale_coul” was set to 1, the “scale_HB” was set to 0.1, and the “scale_tether” was set to 1. The “diel_const” was set to 1 and the “dis_dep_dipole” was set to off.

### Calculation of Binding Energy Value

According to the *InsightII* program, the total interaction energy (Δ*E*_binding_) between the receptor protein and the ligand is usually calculated using the *Evaluate Intermolecular Energy* function of the *Docking* module. The Δ*E*_binding_ value includes two components, namely, the van der Waals (VDW) interaction energy (Δ*E*_VDW_) and the Coulomb interaction energy (*i.e.*, electrostatic interaction energy, Δ*E*_Coulomb_). Hydrogen bond energy is included as part of the Coulomb interaction energy. The following equation 1 is commonly used to calculate the total interaction energy Δ*E*_binding_:
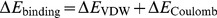
(1)


Theoretically, Δ*E*_binding_ can be used to reflect the experimentally determined log*RBA,* as expressed in equation 2 below:
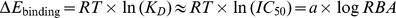
(2)


Here a higher value of Δ*E* would reflect a lower binding affinity. A higher degree of correlation between Δ*E*_binding_ and log*RBA* would indicate that the computational docking model has a higher degree of accuracy to predict a compound’s relative binding affinity for the receptor.

## Results and Discussion

### Validation of the Molecular Docking Method

**Figure 2:**
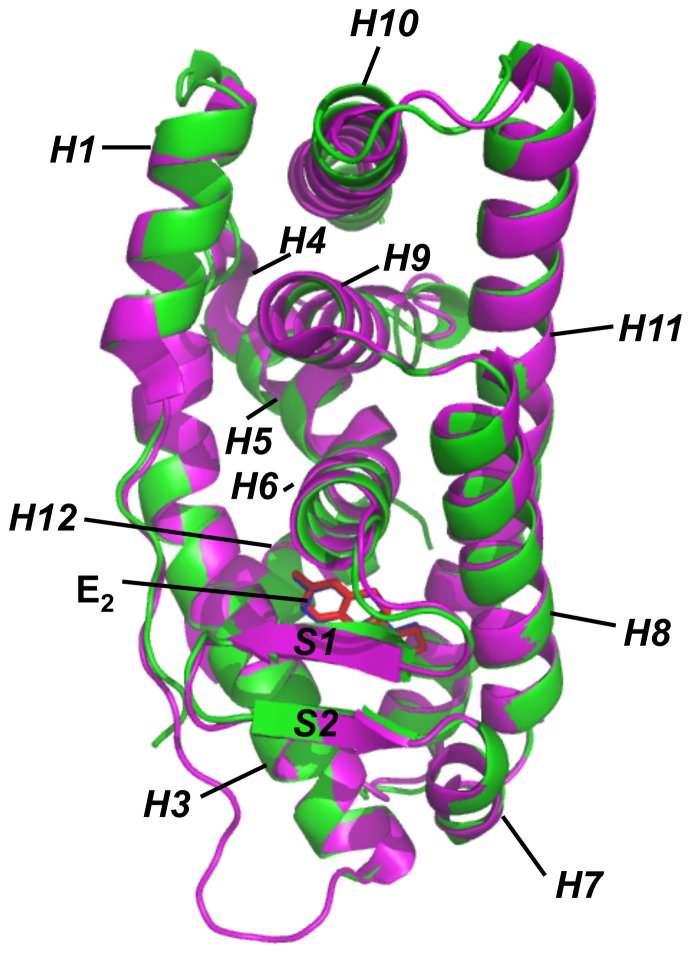
The overlay of the ligand-binding domains (LBDs) of ERα and ERβ. The protein structures were shown in cartoon and colored green and magenta for ERα and ERβ, respectively. E_2_ molecules were shown in stick and colored blue and red in ERα and ERβ LBD, respectively. α-Helixes and β-sheets in the ER LBDs are labeled according to references [14], [16]. Helix 2 structures are missing in both *X*-ray structures.

Consistent with an earlier report [19], when the structures of the ERα and ERβ LBDs are superimposed to each other (shown in **Figure 2**), the basic folds of their LBDs are very similar to each other, and the overall positions of the binding pockets, as well as the ligand conformation, are nearly identical. Based on this information, next we chose to use the ERα LBD as a representative example for validation of the computational methods used in this study.

The 3-D structure of the ligand binding domain (LBD) of human ERα in complex with DES, a non-steroidal estrogen, has been previously determined (PDB code: 3ERD). To determine whether the computational docking approach can produce the same binding mode for DES as observed in the crystallographic study, we performed a cross-docking validation by using the crystal structure of the human ERα LBD in complex with E_2_ (PDB code: 1ERE) as a template receptor for the docking of DES. The docking model of the ERα-DES complex was then compared with the known crystal structure of the ERα-DES complex (PDB code: 3ERD) by superimposing these two structures.

**Figure 3:**
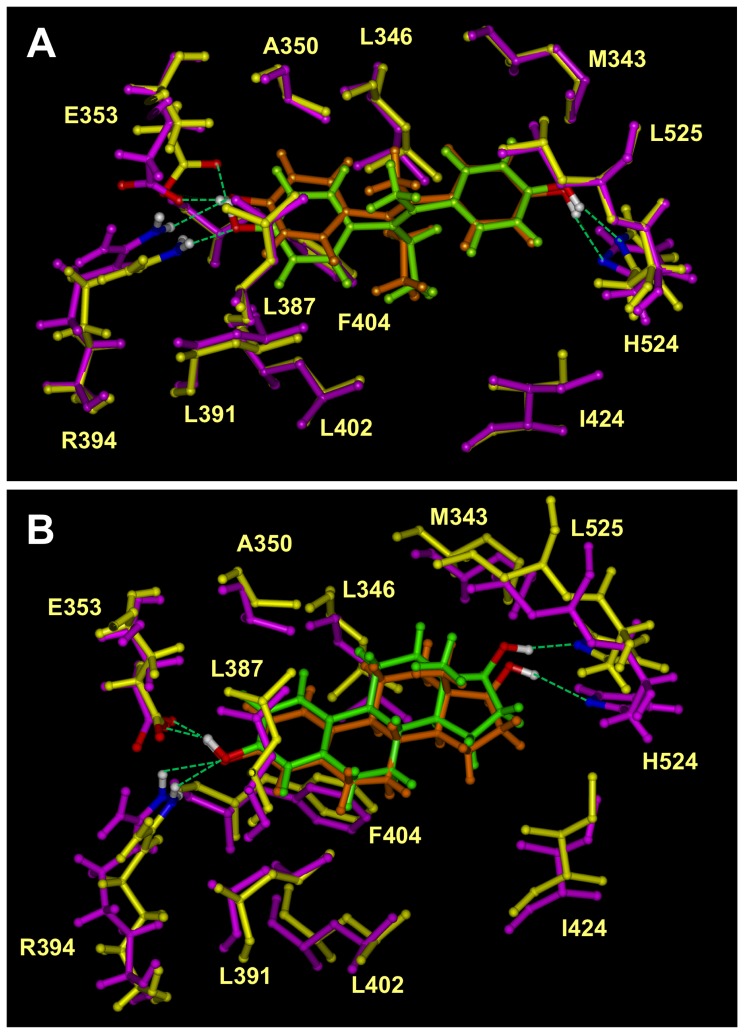
Overlay of docked and crystal structures of ERα LBD in complex with DES or E_2_. A . Superimposed structures of docking result and the crystal structure of ERα LBD in complex with DES. The known crystal structure of ERα LBD in complex with DES (PDB code: 3ERD) was colored in yellow with DES colored in green. The docked DES was colored in orange and ERα LBD was colored in magenta. **B**. Superimposed structures of docking result and the crystal structure of ERα LBD in complex with E_2_. The known crystal structure of ERα LBD in complex with E_2_ (PDB code: 3ERE) was colored in yellow with E_2_ colored in green. The docked E_2_ was colored in orange and ERα LBD was colored in magenta. The green dashes indicated the hydrogen bonds formed. All the structures were shown in ball and stick. The atoms involved with hydrogen bond formation were colored according to the atom type, i.e. white for hydrogen, red for oxygen and blue for nitrogen. Hydrogen atoms in other amino acids were not shown.

As shown in **Figure 3A**, the docked structure of ERα-DES complex has a very similar overall structure as the known 3-D structure of this complex. The overall binding conformation of the modeled DES ligand is almost identical to its experimentally derived structure with the structures showing that the two aromatic rings of DES have the same steric orientation, and form four hydrogen bonds with residues E353, R394 and H524 in the binding pocket. These three amino acids have less than 0.4 Å RMSD between the docked structure and the crystal structure. In addition, several other important residues in the binding pocket, namely, A350, L346, M343, L525, I424, L402, L391, F404 and L346, form almost identical hydrophobic interactions with DES in both modeled and crystal structures.

Similarly, we used the known crystal structure of ERα LBD that was complexed with DES (PDB code: 3ERD) as a template and then docked E_2_ into the LBD. The docking results were compared with the known crystal structure of the ERα-E_2_ complex (PDB code: 1ERE). As shown in **Figure 3B**, the docking method produced a nearly identical structure of the ERα-E_2_ complex compared to the known 3-D structure of this complex. Three identical hydrogen bonds are identified in both docked and known crystal structures, which are formed between the two hydroxyl groups of E_2_ and the amino acid residues E353, R394 and H524 in the binding pocket. Also, all hydrophobic amino acids in the binding pocket have nearly the same orientation and positioning in these two structures. Together, these two examples show that the docking method used can correctly predict the binding mode of a ligand in the LBDs of human ERs with a high degree of reliability and accuracy.

### Prediction of the Binding Mode of E_2_ Derivatives in the LBDs of Human ERα and ERβ

By using the same docking approach as described above, next we docked a total of 27 E_2_ derivatives into the LBDs of human ERα and ERβ. These 27 analogs were selected from a pool of some 50 estrogen derivatives tested in our earlier study [11]. They were divided into three groups according to their structural relationship with E_2_ (see **Table 1**): *A*-ring derivatives (9 compounds including E_2_ itself), *B*/*C*-ring derivatives (8 compounds), and *D*-ring derivatives (10 compounds). These 27 ligands all share the same core structure as E_2_ with only one or two small functional group(s) being added to the steroid core (see **Figure 1**). The purpose of the docking study was to determine how these subtle modifications in the ligand structures alter the binding conformations and binding energy values (which reflect the binding affinity) of these ligands.

#### *A*-ring derivatives

**Figure 4:**
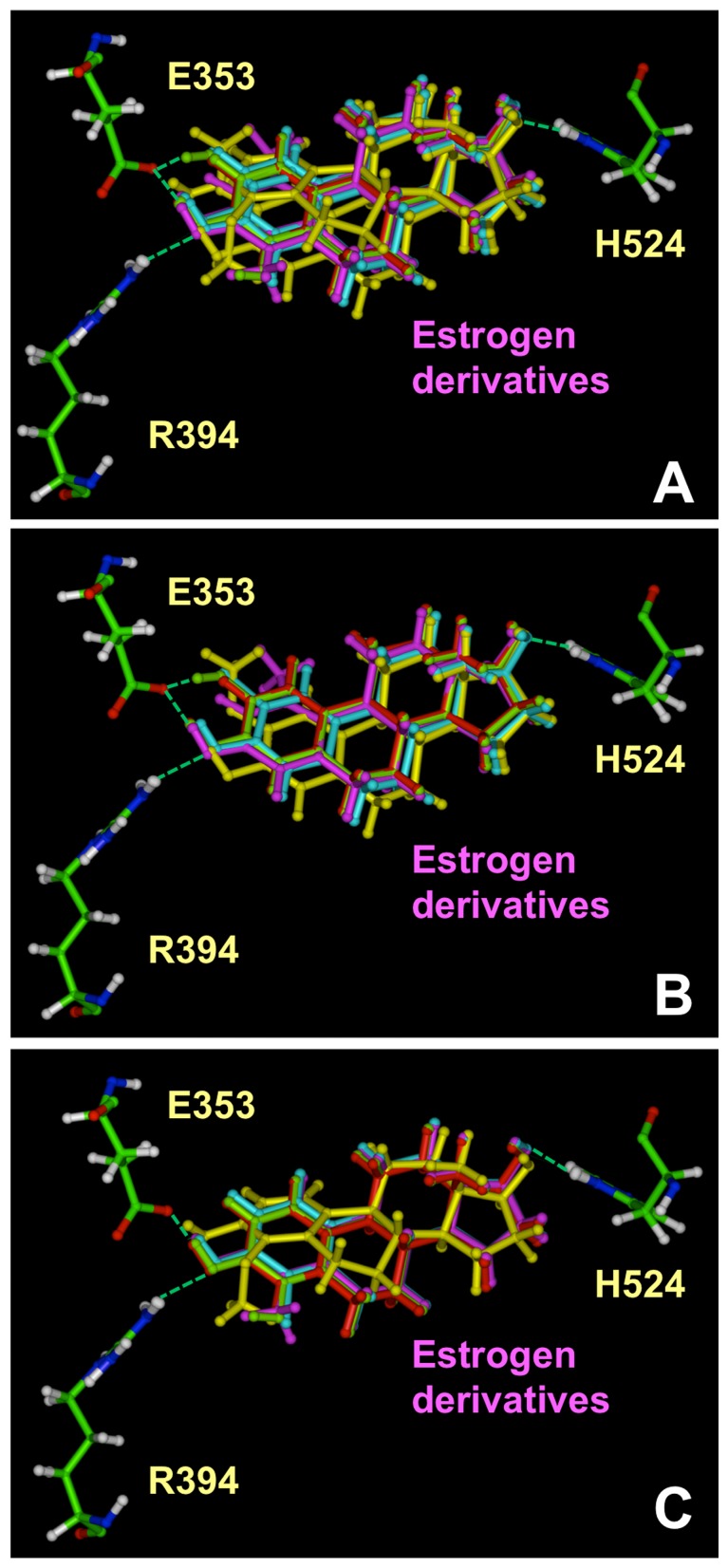
Interactions of *A*-ring derivatives with ERα LBD determined by the molecular docking method. The green dashes indicate the hydrogen bonds formed. All the structures are shown in ball and stick. The amino acids were colored according to the atom type, i.e. green for carbon, red for oxygen, blue for nitrogen and white for hydrogen. Among the amino acids in the binding site, only E353, R394 and H524 were shown in this figure. E_2_ was colored in red; 1-methyl-E_2_ and 4-Methyl-E_2_ were colored in magenta; 2-OH-E_2_ and 4-OH-E_2_ were colored in green; 2-Br-E_2_ and 4-Br-E_2_ were colored in blue; 2-MeO-E_2_ and 4-MeO-E_2_ were colored in yellow. **A**. Overlay of all the *A*-ring derivatives. **B**. Overlay of E_2_, 1-methyl-E_2_, 2-MeO-E_2_, 2-OH-E_2_ and 2-Br-E_2_. **C**. Overlay of E_2_, 4-methyl-E_2_, 4-MeO-E_2_, 4-OH-E_2_ and 4-Br-E_2_.

The overall 3-D binding modes of these *A*-ring derivatives were very similar to that of E_2_. Two hydrogen bonds are formed between their 3-hydroxyl groups and the amino acid residues E353 and R394 in ERα or E305 and R346 in ERβ. A third hydrogen bond was formed between the 17-hydroxyl group of E_2_ and ERα-H524 or ERβ-H475. In the ligand-bound structures that were modeled, other hydrophobic amino acids in the binding pocket had a similar conformation to E_2_ **.** The docking results of various *A*-ring derivatives of E_2_ with ERα LBD are shown in **Figure 4**.

For most of the *A*-ring derivatives, there were only subtle differences between their binding modes in comparison with the binding mode of E_2_. Apparently, these small differences in the binding modes contribute importantly to their differential binding affinities for the ERs. One of the most notable differences in the binding of these *A*-ring estrogen derivatives was that the hydrogen bonds formed with their 3-hydroxyl group were of slightly different length. It is hypothesized that the difference in the hydrogen bond distance, which would affect the hydrogen bond strength, is an important determinant of the binding affinity of a given ligand. To test this hypothesis, we computed for comparison the distances of the following three hydrogen bonds formed between the 3-hydroxyl group of various estrogens: the hydrogen bond between the H atoms of the 3-hydroxyl group and the O_ε_ of ERα-E353 or ERβ-E305, the hydrogen bond between the O atom of the 3-hydroxyl group and the H_η_ of ERα-R394 or ERβ-R346, and the hydrogen bond between the H atom of the 17-hydroxyl group of estrogens and the N_δ_ of ERα-H524 or ERβ-H475. The values are summarized in **Table 1**. To decipher the contribution of each hydrogen bond to the overall binding affinity, we determined the degree of correlation between the hydrogen bond lengths with the experimentally determined log*RBA* values. Given that the hydrogen bond interactions are generally thought to be electrostatic interactions in nature and that the electrostatic potential is usually in an inverse first-order relationship with the distance between the two atoms, the inverse first-order curve regression was thus used here to correlate the experimentally determined log*RBA* values with the hydrogen bond length.

**Figure 5:**
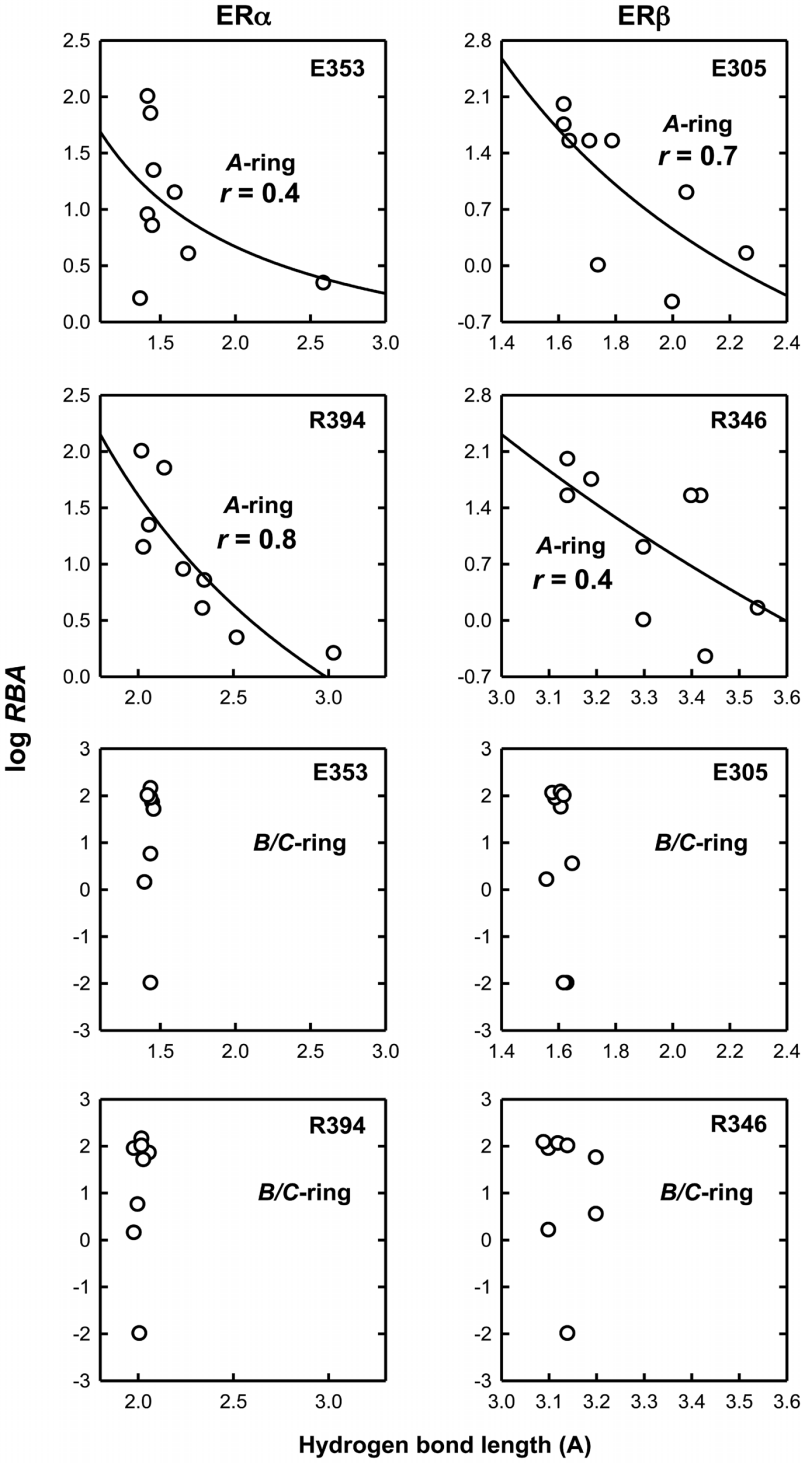
Correlation of hydrogen bond length and log*RBA* of *A*-ring and *B*/*C*-ring derivatives. The hydrogen bond length data were shown in **Table 1**. The amino acids shown in the up right corner of each indicated that the hydrogen bonds were formed between 3-hydroxyl groups of the *A*-ring or *B*/*C*-ring derivatives and this specific amino acid in the binding pocket. The curve regression was performed according to the Inverse First Order equation y = y_0_+ a/x.

For the *A*-ring derivatives, two hydrogen bonds are formed between the 3-hydroxyl groups of these estrogen derivatives and the amino acid residues E353 and R394 in ERα or E305 and R346 in ERβ. As shown in **Figure 5**, for ERα, the inverse correlation for the distance of the hydrogen bond with R394 is better than that for E353, suggesting that the hydrogen bond formed between the 3-hydroxyl group of various estrogens and R394 plays a more important role in the binding interaction than the hydrogen bond formed with E353. For ERβ, the correlation for the length of the hydrogen bond formed with E305 is stronger than that for R346, again suggesting that these two hydrogen bonds contribute differentially to the binding affinity. The substantial correlation between the experimentally determined log*RBA* values and the hydrogen bond lengths indicates the vital importance of the hydrogen bonds with the 3-hydroxyl group of E_2_ in determining their overall binding affinity of a ligand. This correlation also suggests that computing the lengths of the hydrogen bonds formed between the 3-hydroxyl group of a steroidal estrogen and ERs can be used as an important predictor of the binding affinity of a given *A*-ring derivative of E_2_. The hydrogen bonds formed between the 17-hydroxyl group of *A*-ring derivatives of E_2_ with H524 in ERα or H475 in ERβ were of similar lengths and less important to determine their binding affinities.

Earlier studies suggested that 4-OH-E_2_ has a markedly lower dissociation constant for the ERs compared to E_2_, which may mean that 4-OH-E_2_ can remain in the ERα LBD longer [12], [20]. Our computational docking model showed that 4-OH-E_2_ could form an ideal hydrogen bond between its 3-hydroxyl group and E353 and R394 in the same way as seen with E_2_. In addition, 4-OH-E_2_ can also form a hydrogen bond between its 4-hydroxyl group and ERα-L387. In the case of ERβ, a similar hydroxyl group is also formed with L339. This interaction is believed to account for its slower dissociation with the ERs. In this context, it is also of note that our docking results show that 2-OH-E_2_ can also form an additional hydrogen bond between its 2-hydroxyl group and ERα-E353 or ERβ-E305. Interestingly, despite the presence of additional hydrogen bond, 2-OH-E_2_ actually has a lower binding affinity for both ERα and ERβ compared to E_2_. Our docking model of 2-OH-E_2_ provides unique insights. The presence of an additional 2-hydroxyl group causes a unfavorable interaction with the hydrogen bond formation between its 3-hydroxyl group and ERα-E353 or ERβ-E305, which likely is the main cause that reduces its overall binding affinity for the ERs.

Among the *A*-ring derivatives tested, 2-MeO-E_2_ and 4-MeO-E_2_ caused the most drastic shift of the ligand position relative to E_2_ due to their bulkier methoxy substituents, which shifts the ligand position and results in less favorable van der Waals interactions and also interferes with the formation of hydrogen bonds between their 3-hydroxyl groups and ERα-E353 and R394 or ERβ-E305 and R346, ultimately resulting in drastically reduced binding affinities.

#### *B*/*C*-ring derivatives

**Figure 6:**
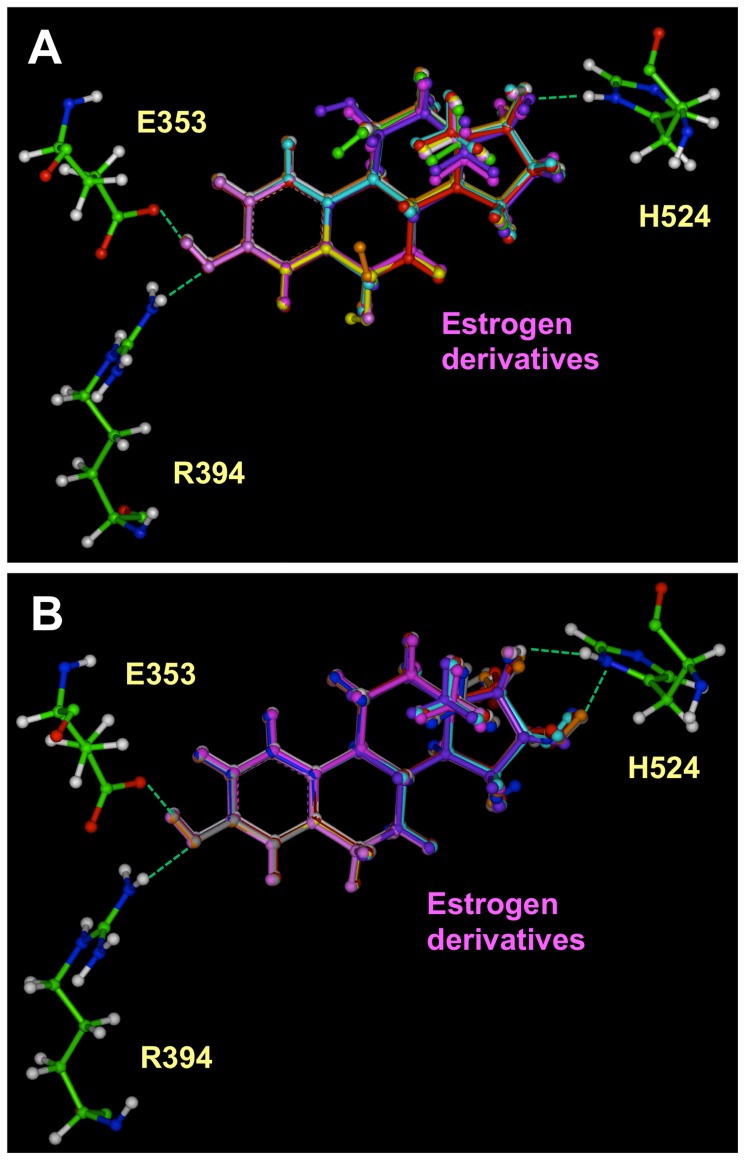
Interactions of *B*/*C*-ring (A) and *D*-ring derivatives (B) with ERα LBD determined by molecular docking. The green dashes indicate the hydrogen bonds formed. All the structures are shown in ball and stick. The amino acids were colored according to the atom type, i.e. green for carbon, red for oxygen, blue for nitrogen and white for hydrogen. Among the amino acids in the binding site, only E353, R394 and H524 are shown in this figure. E_2_ was colored in white. The ligands were shown in the following different colors: in **panel A**, 6α-OH-E_2_ (yellow), 6β-OH-E_2_ (orange), 6-keto-E_2_ (pink), 6-dehydro-E_2_ (red), 7-dehydro-E_2_ (magenta), 9(11)-dehydro-E_2_ (light blue), 11α-OH-E_2_ (purple) and 11 β-OH-E_2_ (green); in **panel B**, E_1_ (magenta) estriol (16α-OH-E_2_) (yellow), 16β-OH-E_2_ (orange), 16-keto-E_2_ (pink), 17α-OH-E_2_ (red), 15α-OH-E_3_ (dark blue), 16α-OH-E_1_ (light blue), 16-keto-E_1_ (purple), 16α-OH-E_2_-17α (brown), 16β-OH-E_2_-17α (grey).

The docking results of various *B*/*C*-ring derivatives with the LBDs of ERα and ERβ are shown in **Figure 6** **.** Similar to *A*-ring derivatives, we noticed that their binding modes are very close to that of E_2_. Two hydrogen bonds are formed between the 3-hydroxyl group and residues E353 and R394 in ERα or E305 and R346 in ERβ. The lengths of their hydrogen bonds are similar to those formed with E_2_. No hydrogen bonds were observed with either the 6- and 11-hydroxyl groups of the *B*/*C*-ring derivatives in the modeled structures.

For *B*/*C*-ring derivatives, the correlation coefficient *r* values between their hydrogen bond lengths and log*RBA* values are 0.1, 0.2 and 0.4 for the hydrogen bonds with E353, R394 and H524 of ERα, respectively (**Figure 5**). For ERβ, the *r* values are 0.3, 0.1 and 0.4 for the hydrogen bonds with E305, R346 and H475, respectively. The poor correlations for the *B*/*C*-ring derivatives indicate that the binding interactions contributed by the 3- and 17β-hydrogen bonds are not the determining forces of the binding affinity of the *B*/*C*-ring derivatives. As discussed below, the repulsive forces play a dominant role in determining the binding affinity of *B*-ring derivatives.

#### *D*-ring derivatives

For some of the *D*-ring derivatives with a hydroxyl group at the C-16 position, one more hydrogen bond can be formed with H524, which may contribute to their relatively higher binding affinity for the ERs as compared to other estrogen metabolites (**Figure 6**). The correlation coefficient *r* ^2^ values for the *D*-ring derivatives are 0.13, 0.06 and 0.04 for the hydrogen bonds observed with E353, R394 and H524 of ERα, respectively (hydrogen bond lengths were shown in **Table 1**; correlation data were not shown). For ERβ, the *r* ^2^ values are 0.00 (no correlation at all), 0.00 and 0.08 for the hydrogen bonds with E305, R346 and H475, respectively. For the *D*-ring derivatives that form two hydrogen bonds with histidine, only the shorter hydrogen bond was used in the correlation. Similarly to the *B*/*C* ring derivatives, the poor correlation for the *D*-ring derivatives suggested that the binding interactions contributed by the 3 and 17β hydrogen bonds are not the determining force of the binding affinity of the *D*-ring derivatives. The role of repulsive forces in determining the binding affinity of *D*-ring derivatives is discussed later.

### Prediction of Binding Affinity of Estrogen Derivatives with ERα and ERβ LBDs Based on Binding Energy Calculation

**Figure 7:**
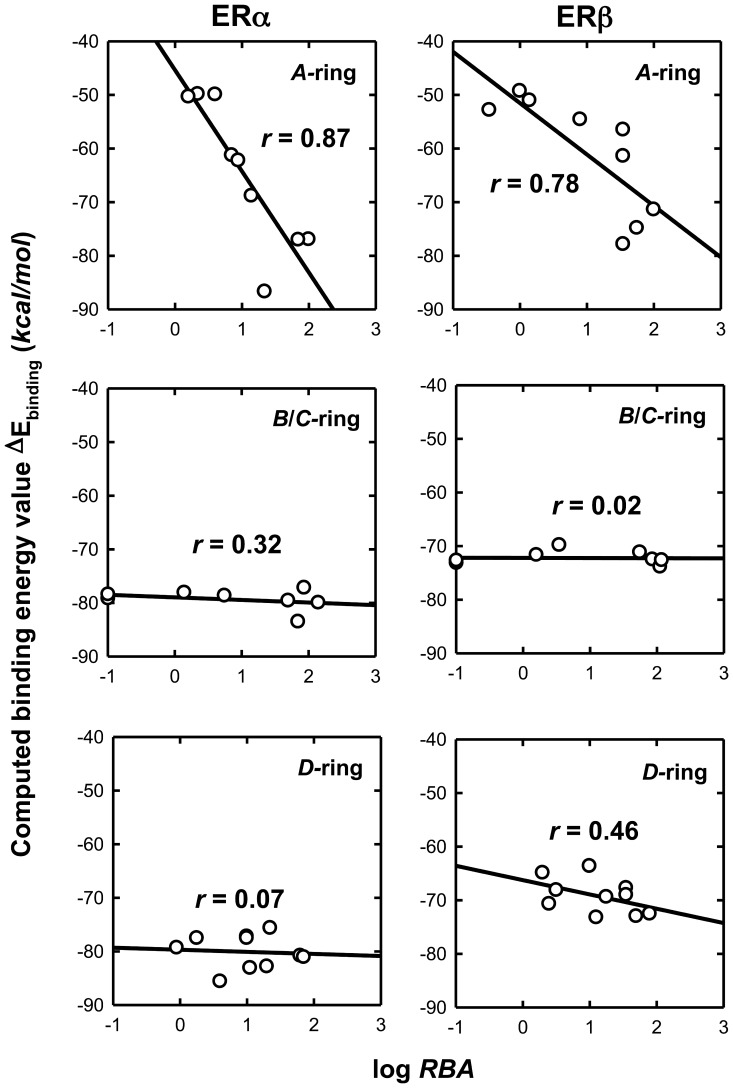
Correlation between log*RBA* and binding energy calculated with equation (1) and data in Table 1. The correlation coefficient *r* value is shown in the figure.

According to the docking models developed in this study for each of the 27 E_2_ derivatives, we have also computed their binding energy (Δ*E*) values in complex with ERα or ERβ (listed in **Table 1**). The correlation between the computed Δ*E*_binding_ values and the experimentally determined log*RBA* values for various E_2_ derivatives is shown in **Figure 7**. For both ERα and ERβ, the correlation is highest for *A*-ring derivatives, with *r* values 0.87 and 0.78, respectively. In contrast, there was no meaningful correlation seen between the computed values and experimental values for the *B*/*C*-ring derivatives or *D*-ring derivatives.

The poor correlations between the calculated binding energy values and the experimental values for *B*/*C*-ring derivatives or *D*-ring derivatives are, in part, due to the inherent problems associated with the currently-used molecular docking method [21], namely, the relative inaccuracy of the molecular mechanics approach for computing the interaction energy levels. Because of the hydrophilic substitutions (*i.e.*, the hydroxyl group) in their C-6 or C-11 position, both of which are surrounded by hydrophobic amino acid residues, these hydroxyl groups are expected to reduce the chances for their binding interactions with the receptors because of the presence of strong repulsive forces. This low correlation also reveals a major deficiency of the currently used computational program, which cannot properly weigh the role of the repulsive forces that may completely diminish the chances for effective binding interactions.

**Figure 8:**
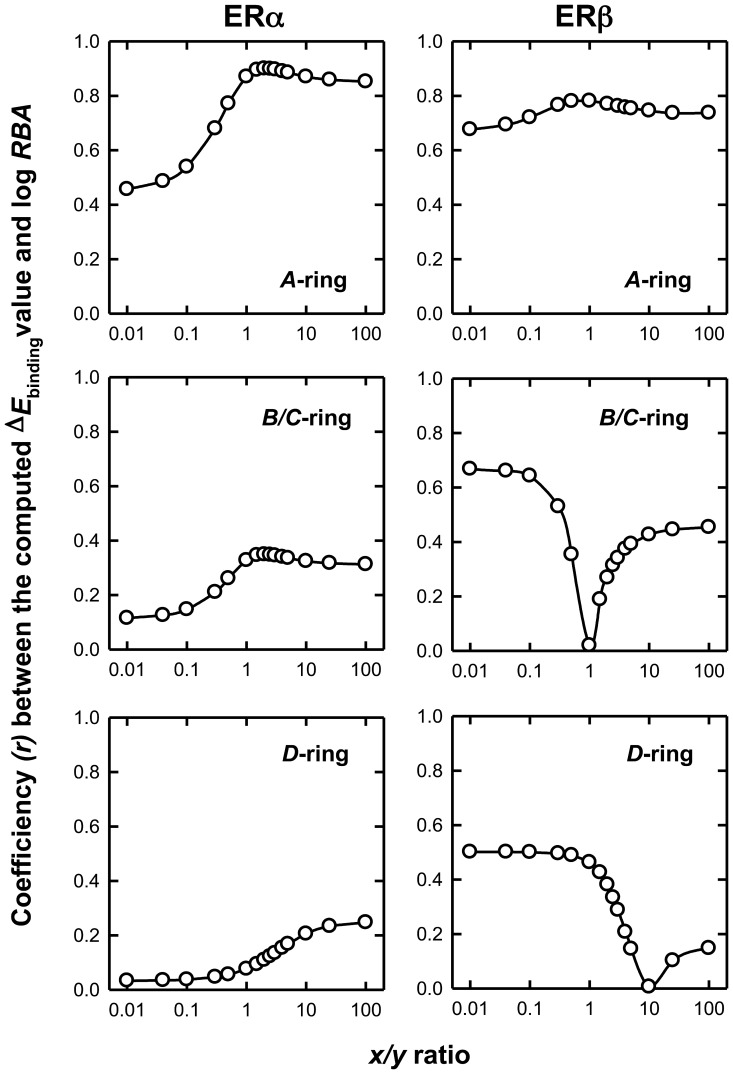
Relationship between the correlation coefficient *r* and *x*/*y* for the estrogen derivatives in Table 1. For each *x*/*y* value, the total binding energy was calculated according to equation (3) and the correlation coefficient *r* value is calculated by correlating the total binding energy for each chemical with its log*RBA*. The *x*/*y* value is shown in log scale.

In an attempt to improve the modeling program to better access the repulsive force, we sought to modify the relative weight of VDW interaction energy (Δ*E*_VDW_) and Coulomb interaction energy (Δ*E*_Coulomb_) in computing the final interaction energy (Δ*E*_binding_), by converting the commonly used equation 1 to the following equation 3:
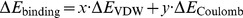
(3)where *x* and *y* are assigned parameters to adjust the relative weights of Δ*E*_VDW_ and Δ*E*_Coulomb_ in the total binding energy. Using the above equation, by altering the *x*/*y* ratios, we can compute the corresponding *r* value for the relationship between the computed Δ*E*_binding_ and experimentally-determined log*RBA*. The relationships between *x*/*y* value and *r* value for *A*-ring, *B*/*C*-ring and *D*-ring derivatives are shown in **Figure 8** **.**

The optimal *r* value was achieved with different *x*/*y* values for *A*-ring and *B*/*C*-ring derivatives. For *A*-ring derivatives, in the case of ERα, when *x*/*y* is 2, the optimal *r* value is 0.9. For ERβ, the curve is basically flat, which means that VDW and Coulomb interaction energy are equally important to determine the *RBA* of a ligand. For *B*/*C*-ring derivatives with ERβ, the optimal *r* is 0.8 when *x* is 0, which indicates that Coulomb energy is more important in determining *RBA*. Interestingly, when *x*/*y* is 1, the correlation is the worst. However, for *B*/*C*- and *D*-ring derivatives, the prediction is still very poor for both ERs. It is apparent that the inability of the docking program to accurately calculate the binding energy value for the *B*/*C-* and *D*-ring derivatives is not simply due to the inappropriate balance of the electrostatic interaction and VDW interaction. Further studies are warranted to better resolve this problem.

Conclusions and practical implicationsThe present study sought to use the molecular docking approach to characterize the interaction of E_2_ derivatives with human ERα and ERβ. First, we tested the suitability of the molecular docking method for predicting the correct binding mode of a ligand inside the binding pockets of human ERα and ERβ. Using DES and E_2_ as examples, we demonstrated that the docked structures are almost exactly the same as the known crystal structures of ERα LBD in complex with either of these two ligands. Using the same docking approach, we also docked 27 structurally-similar E_2_ derivatives into the LBDs of human ERα and ERβ. While their binding modes are very similar to that of E_2_, there are notable subtle differences. These small differences are believed to contribute importantly to their different ER binding affinity. In the case of the *A*-ring estrogen derivatives analyzed, there is a strong inverse relationship between the length of the hydrogen bonds formed with ERs and the binding affinity. In the study, we optimized our approach’s ability to predict the relative binding affinity of a given *A*-ring derivative of E_2_ through re-adjusting the relative contribution of the van der Waals (VDW) interaction energy and Coulomb interaction energy in computing the overall binding energy (Δ*E*) value.

However, for *B*/*C* and *D*-ring estrogen derivatives, the correlations between calculated binding energy and experimentally determined binding affinity is very low and re-adjustment of the relative contribution of the van der Waals (VDW) interaction energy and the Coulomb interaction energy did not improve their correlations. Notably, some recent studies have also noted that while the docking programs can be successfully used to predict the ligand poses, they appear to lack the ability to accurately predict the ligand binding affinity [23], [24]. The inability of the docking programs to predict the binding affinity likely is mainly due to their lack of adequate consideration of the repulsive forces between the hydrophilic substitutions at the C-6 and C-11 positions with the hydrophobic residues around these regions in the docking approach. In addition, it is also rather problematic to account for the desolvation of the hydrophilic groups on the ER ligands as well as in the receptor binding pocket, which may also be a partial contributing factor. Methodological improvements in these particular areas may greatly enhance the docking and scoring approaches, and ultimately, the accuracy in calculating the binding energy values between a ligand and its binding protein.
